# Fabrication and Characterization of Anionic Composite Membranes Produced by Electrospinning Method

**DOI:** 10.3390/polym17121677

**Published:** 2025-06-17

**Authors:** Somayyeh Rakhshani, Rodolfo Araneo, Luis Alexander Hein, Antonio Rinaldi, Alfonso Pozio

**Affiliations:** 1 Department of Chemical Engineering Materials Environment, La Sapienza University of Rome, Via Eudossiana 18, 00184 Rome, Italy; somayyeh.rakhshani@uniroma1.it (S.R.); rodolfo.araneo@uniroma1.it (R.A.); 2 Nanofaber srl, Via Anguillarese 301, 00123 Rome, Italy; luis.hein@nanofaber.com; 3 ENEA, C.R. Casaccia, Via Anguillarese 301, 00123 Rome, Italy

**Keywords:** electrospinning, anion-exchange membrane, water electrolysis

## Abstract

Developing efficient and durable anion-exchange membranes (AEMs) is essential for advancing electrochemical energy technologies such as water electrolyzers. This study presents a methodological approach for fabricating an AEM by electrospinning a polysulfone (PSU)-based nanofibrous matrix, followed by post-activation using an ionomer solution containing quaternary ammonium (QA) functional groups. Electrospinning is a promising and versatile technique for membrane fabrication, particularly in the context of green hydrogen production via AEM water electrolysis. Its ability to produce nanofibrous matrixes with tunable morphology and properties makes it an attractive alternative to conventional methods for research across various applications. This study demonstrated the feasibility of fabricating electrospun AEMs using polysulfone as a backbone material, suggesting its promise as a potentially scalable solution to manage the high-cost issue of commercial AEMs for future hydrogen production. The resulting composite membrane exhibited ionic conductivity and electrochemical performance comparable to a benchmark membrane fabricated by activating a commercial Celgard 3401 support via phase inversion. Although the mechanical strength of the electrospun membrane was lower than that of the commercial support, its good electrochemical characteristics—combined with the potential for roll-to-roll electrospinning—underscore the promise of this approach as a viable, economically scalable strategy for future hydrogen production WE technologies.

## 1. Introduction

**Figure 1:**
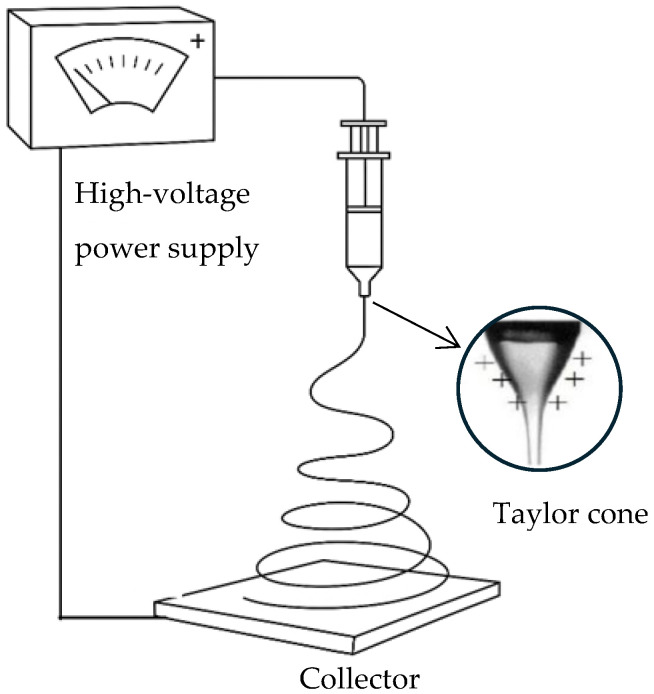
Schematic representation of a typical electrospinning setup.

Electrospinning is a versatile and efficient technique for fabricating fine fibers from a wide range of polymers. The process involves applying high voltage to a polymer solution or melting, which causes the ejection of a charged jet from the tip of a needle or spinneret. As the jet travels towards a grounded collector, it undergoes elongation and solvent evaporation, forming continuous fibers with diameters ranging from nanometers to micrometers. A typical electrospinning setup is illustrated in Figure 1. The simplicity, flexibility, and scalability of electrospinning make it an attractive method for producing nanofibrous materials with high surface area-to-volume ratios and tunable properties [1,2]. Electrospun fibers have found applications in various fields, including biomedicine, filtration, energy storage, and smart textiles, due to their unique properties [3].

The evolution of electrospinning technology reflects a journey from early conceptual experiments to a sophisticated and versatile method for producing advanced materials, with significant impacts across multiple industries [4]. The use of electrospinning in producing anion-exchange membranes (AEMs) has gained significant interest in recent years due to the method’s ability to create membranes with desirable features, such as high surface area, controlled porosity, and tunable fiber morphology. For instance, Samsudin and Hacker developed electrospun quaternary poly(vinyl alcohol) (QPVA) AEMs for fuel cells, demonstrating improved ion-exchange capacity and hydroxide conductivity, highlighting the potential of electrospinning in AEM fabrication [5]. Similarly, Yang et al. compared PVA/CS membranes prepared via solution casting and electrospinning, finding that electrospun composite membranes exhibited improved ionic conductivity and reduced methanol permeability. This underscores the potential of electrospinning to enhance membrane performance in alkaline environments [6]. Shang et al. further reviewed the benefits of electrospun nanofiber composite membranes for both proton- and anion-exchange systems, emphasizing their enhanced mechanical and transport properties [7] Despite this progress, the application of electrospinning to fabricate AEMs using polysulfone (PSU) as the membrane backbone remains underexplored. Most reported efforts involve modifying commercial porous substrates, such as Celgard, using surface functionalization or impregnation techniques. This limits design flexibility and restricts the tunability of the membrane microstructure and performance.

Water electrolysis (WE) is a process that uses electrical energy to split water into hydrogen and oxygen gases. Among the two primary types—proton-exchange membrane water electrolysis (PEMWE) and anion-exchange membrane water electrolysis (AEMWE)—the latter has emerged as a promising alternative due to its potential use of earth-abundant, non-precious metal catalysts and less corrosive alkaline electrolytes. In AEMWE systems, the membrane must efficiently conduct hydroxide ions (OH^−^) while maintaining chemical and mechanical stability in alkaline environments. These membranes—referred to as hydroxide-exchange membranes (HEMs)—are critical to ensuring the performance, efficiency, and durability of the electrolyzer.

**Figure 2:**
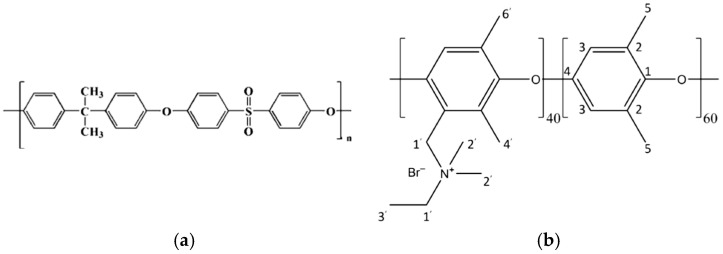
(**a**) Chemical structure of PSU; (**b**) the structure of a Fumion ionomer proposed by Giovanelli et al. [19].

Recent studies have emphasized the importance of selecting appropriate polymer backbones for HEMs to ensure their stability and performance in alkaline environments. Several polymer backbones have been investigated for HEM development, including polyethylene, poly(aryl ether)s, polybenzimidazoles, and poly(phenylene oxide) [8,9,10]. However, polysulfone (PSU) stands out due to its favorable combination of thermal stability, mechanical strength, oxidative resistance, and ease of functionalization with quaternary ammonium groups [11,12,13]. Its rigid aromatic backbone (Figure 2a) is particularly robust in alkaline media, making it a reliable platform for developing durable AEMs for electrolysis applications. Additionally, PSU can be electrospun into nanofiber mats, enabling integrated membrane design without reliance on commercial scaffolds [14]. Nevertheless, designing AEMs that achieve high hydroxide ion conductivity while maintaining chemical and dimensional stability remains a significant materials challenge. Enhancing conductivity often compromises other essential properties of the membrane. Furthermore, the synthesis of HEMs involves complex chemical processes to introduce functional groups that facilitate hydroxide ion transport. These processes need to be precisely controlled to achieve the desired membrane properties. Historically, more research and development have been directed towards proton-exchange membranes (PEMs) for water electrolysis due to their earlier adoption and commercial success. Advances in materials chemistry [15], membrane electrode assembly optimization [16], and innovative membrane designs [17] are driving the field forward. Continued research into advanced and cost-effective production methods with high stability and performance of AEMs, which we are following in this study, will be essential for the commercial viability of AEMWE technology. As reported in recent techno-economic analyses of AEMWE for green hydrogen production, further technological advancements and scaling up are critical to unlocking the full potential of this technology. With the continued decline in renewable energy costs and improvements in AEM stack lifetimes, the levelized cost of hydrogen from AEMWE systems is projected to become highly competitive with PEMWE systems [18].

In this study, we present a novel approach to anion-exchange membrane (AEM) fabrication by employing electrospun polysulfone (PSU) as the structural membrane backbone, followed by ionic functionalization using Fumion^®^, a commercially available solution containing a quaternary ammonium (QA)-based ionomer. While the exact chemical composition of Fumion^®^ is not disclosed by the manufacturer, recent work by Giovanelli et al. [19] employed multinuclear SSNMR to elucidate the structure of its functional groups. The proposed molecular structure derived from their analysis is illustrated in Figure 2b of this work.

The performance of the resulting membrane competes with that of its commercial counterpart, which involves the fabrication of a composite anion-exchange membrane by activating a porous support of commercial Celgard 3401 using an ionomer solution in a phase-inversion process [20]. This ongoing research is progressively addressing the challenges associated with the development of anion-exchange membranes. These efforts focus on creating electrochemically efficient and potentially cost-effective AEMs by employing low-cost, processable materials and optimizing fabrication methods. This approach suggests promising potential to support the broader adoption of hydrogen as a clean energy source through water electrolyzer performance.

## 2. Materials and Methods

### 2.1. Materials

PSU granules (Grade: Udel^®^ P 1700 from Goodfellow, Cambridge, UK), NMP (100% purity, VWR, Radnor, PA, USA), Fumion (Fumion^®^ FAA-3, 10% solution in NMP by Fuma-Tech, Stuttgart, Germany), ethanol 96% *v*/*v* (Panreac Quimica Sau, Barcelona, Spain), and toluene (Clean Consult International S.p.a. Milan, Italy) are the materials used in this experiment.

### 2.2. Preparation of the Anion-Exchange Membrane

#### 2.2.1. Preparation of Polysulfone Matrix Membranes

The polymer solution is prominent in electrospinning technology, serving as the foundational material from which nanofibrous structures are fabricated. Polymer solution characteristics such as viscosity, conductivity, surface tension, solvent volatility, and polymer concentration directly influence the electrospinning process by affecting fiber formation, diameter, morphology, and spinnability [21,22,23,24,25,26,27]. Understanding and optimizing the polymer solution characteristics is essential for producing high-quality electrospun membranes tailored for specific applications. The polymer concentration in the solution is a critical determinant of the electrospinning outcome. It affects the viscosity and surface tension, which, in turn, influences the fiber formation process [21,24,27]. Insufficient chain entanglement at low concentrations can lead to beads rather than continuous fibers forming [27]. Conversely, excessively high concentrations result in high viscosities that impede the formation of a stable jet and Taylor cone, leading to defects and irregularities in the fibers. For polysulfone, a 24% *w*/*w* concentration in N-Methyl-2-pyrrolidone (NMP) is identified to be optimal based on previous lab experiments guided by a design of experiments (DOE) approach [28]. Therefore, 32.6 g of PSU granules were pre-dried at 60 °C for 24 h. Subsequently, the dried granules were dissolved in 100 mL of NMP under continuous stirring at 50 °C until complete dissolution was achieved. The electrospun membrane was synthesized using an electrospinning machine (Fluidnatek LE100, Bioinicia SI, Valencia, Spain), which is equipped with a large stationary planar metallic collector (x–y plane) underneath a single scanning emitter that can be driven in both the x and y directions, at a controllable speed, to cover a desired deposition area on the collector. The distance between the collector and emitter along the *z*-axis is also adjustable. The emitter and the collector are connected to their high-voltage generator (V_i_ = injector voltage and V_c_ = collector voltage). The polymer solution was fed into the emitter at an adjustable rate controlled by a syringe pump. The second group of parameters that are important in electrospinning technique are the processing parameters, including the applied voltage (ΔV) that determines the electrostatic force driving the jet. Too low a voltage may not overcome the surface tension, while too high a voltage can cause excessive jet instability. The rate at which the polymer solution is fed to the spinneret (flow rate) affects the fiber diameter and uniformity. An optimal flow rate (FR) ensures a stable jet without dripping or breaking. The distance between the spinneret and the collector (d) influences the flight time and solvent evaporation. Typical distances range from 10 to 20 cm, providing sufficient time for solvent evaporation and fiber solidification. The inner diameter of the spinneret, which is called the needle gauge, affects the initial jet formation. Smaller diameters reduce the likelihood of clogging and produce finer fibers with fewer defects. The final critical factors to be controlled in electrospinning are the environmental conditions, specifically the temperature (T) and relative humidity (RH). These parameters significantly impact solvent evaporation and fiber morphology. Elevated temperatures can accelerate solvent evaporation, promoting rapid solidification of the fibers. Conversely, higher humidity levels can result in thinner fibers due to increased interaction with water vapor, which affects the solvent evaporation rate and fiber formation process. We also find that controlling environmental conditions during the drying process is crucial. For instance, when the electrospinning process occurs in high humidity and the resulting membrane is removed from the machine into an ambient environment with significantly lower humidity, it slows down the drying process. This causes the fibers to fuse together, resulting in a nonporous film instead of the intended flat, fibrous sheets.

#### 2.2.2. Preparation of the Ionomer Solution

**Figure 3:**
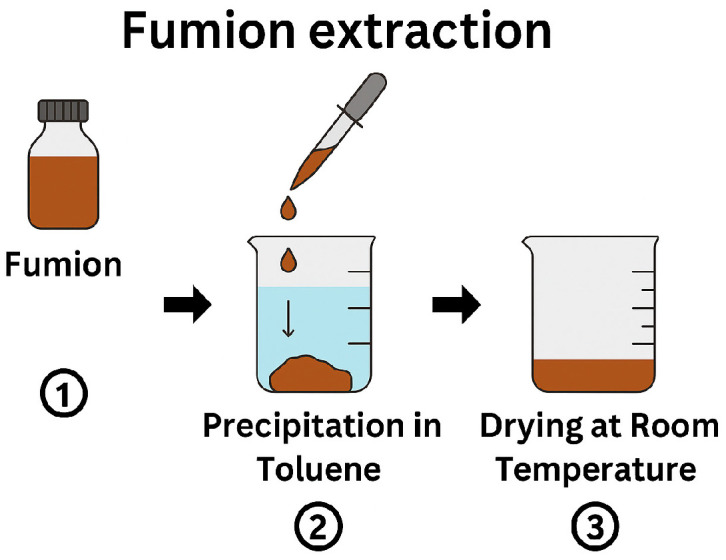
Schematic representation of the Fumion extraction process.

To prepare an ionomer solution suitable for functionalizing PSU membranes, we utilized a commercial ionomer solution, Fumion, which is a 10% solution in N-Methyl-2-pyrrolidone (NMP). The direct use of this solution was not feasible due to its propensity to dissolve the PSU membrane. To circumvent this issue, we extracted the Fumion polymer via precipitation from the commercial solution using toluene. The procedure involved the dropwise addition of the Fumion solution into a small beaker containing toluene, resulting in the rapid precipitation of Fumion. The excess toluene was then decanted, and the precipitated Fumion was allowed to dry at room temperature for several days (Figure 3). Subsequently, the dried Fumion was dissolved in ethanol to prepare ionomer solutions of varying concentrations (Fu 5%, Fu 10%, Fu 15%, and Fu 20% *w*/*w*) for the functionalization of the PSU membrane.

The electrospun PSU membrane was then cut into a 4 cm × 4 cm piece. During the activation process, 0.5 mL of the ionomer solution is applied to the first surface of the sample, and the ionomer solution penetrates inside the fibers network and closes the structure. The membrane was then left to sit for 4 min, allowing the excess solution to drain off by tilting the dish. The same procedure was subsequently repeated on the second surface of the membrane. After both sides were treated, the membrane was left to dry at room temperature.

### 2.3. Characterization

Characterization was conducted using a previously described procedure [29]. This involved examining the morphology of the electrospun samples using a field-emission gun scanning electron microscope (LEO1530, ZEISS, Jena, Germany) and measuring the sample thickness (δ) with a Digital Centesimal Comparator (KATSU). The device operates by determining the distance between a flat reference base and a movable probe that gently contacts the surface of the sample. The thickness reported for each sample is the average thickness of 5 points on 16 cm^2^ surface area.

Air permeability measurement was conducted using Gurley Precision Instruments 4118 (Troy, NY, USA), which measures the time (t) taken for a specific volume of air to pass through a defined surface area (A) under uniform pressure [30].

The membrane’s ability to conduct ions was determined by assessing membrane resistance using an H-cell kit (Scribner, LLC, Southern Pines, NC, USA). This cell consists of two compartments, each featuring two sections for platinum electrodes and reference electrodes. The compartments are sealed by two gaskets, ensuring watertight housing for membranes with a diameter of 3.6 cm and effectively separating the two compartments. This configuration exposes a surface area of 7.07 cm^2^ to the solution, facilitating the flow of electric current between the platinum electrodes and through the membrane. Ionic conductivity measurements were conducted between two Ag/AgCl reference electrodes.

The membrane’s ionic conductivity (σ) was determined by conducting a series of measurements, each involving the determination of the potential difference between the reference electrodes. By applying a linear voltage sweep at brief intervals (every 5 s), a response of voltage versus current was generated, allowing for the determination of resistance (Ω) from the slope of the fitted line. Having resistance, the conductivity (σ) is calculated. This measurement was carried out 6 times for each sample, and the average value is reported.

Ultimately, the mechanical properties of the produced sample underwent assessment through uniaxial tensile testing with a dynamic mechanical analyzer, Discovery DMA 850 (from TA instrument, New Castle, DE, USA) at ambient temperature. Young’s modulus (E) is determined by calculating the slope of the stress–strain curve in the elastic region, reflecting the material’s stiffness, and the ultimate tensile strength (UTS) is determined by identifying the maximum stress the material can withstand before failure, reflecting the material’s strength.

The electrochemical performance of the membrane was evaluated using a lab-scale two-electrode electrolyzer with an active area of 2.0 cm^2^, constructed entirely of steel, operated at room temperature. Stainless-steel gas diffusion electrodes (GDEs, Ø 16 mm) supplied by Bekaert Fiber Technologies (Zwevegem, Belgium) were used on both the anodic and cathodic sides. These GDEs provided electrical contact with the anion-exchange membrane and facilitated the diffusion of water and generated gases. A 0.5 M KOH solution was continuously circulated on the anodic side at a flow rate of 100 mL min^−1^. The cathodic outlet could be connected to a volumetric system for hydrogen quantification. Electrochemical characterization was conducted using a Solartron 1287 potentiostat/galvanostat and a Solartron 1260 frequency response analyzer, both controlled via a GPIB interface. Electrochemical impedance spectroscopy (EIS) was carried out in the frequency range of 1 MHz to 1 Hz at open-circuit potential (OCP), using a 10 mV peak-to-peak AC signal. Static and dynamic galvanic polarization tests were also performed using the same cell setup. Voltage–current (E–I) curves were recorded at 298 K across a current density range of 0–1000 mAcm^−2^. Voltage–time profiles and periodic impedance spectra were acquired throughout the experiments. EIS evaluates a system’s resistance to alternating current and reflects both kinetic and mass transport phenomena. Impedance data is presented as Nyquist plots, where the high-frequency intercept (R_HF_) on the real axis represents the system’s ohmic resistance. The semicircular arc diameter corresponds to the polarization resistance arising from charge transfer and diffusion. Experiments were conducted in a 0.5 M KOH flow at 25 °C. From R_HF_, the specific resistance (R_Ω_ in Ωcm^2^) was calculated using R_Ω_ = R_HF_ × S, where S is the membrane area (2.0 cm^2^). This also enabled the estimation of the membrane’s intrinsic ionic conductivity, which depends on the presence of counter-ions and the hydration level.

## 3. Results and Discussions

### 3.1. Electrospinning Polysulfone Membranes

**Figure 4:**
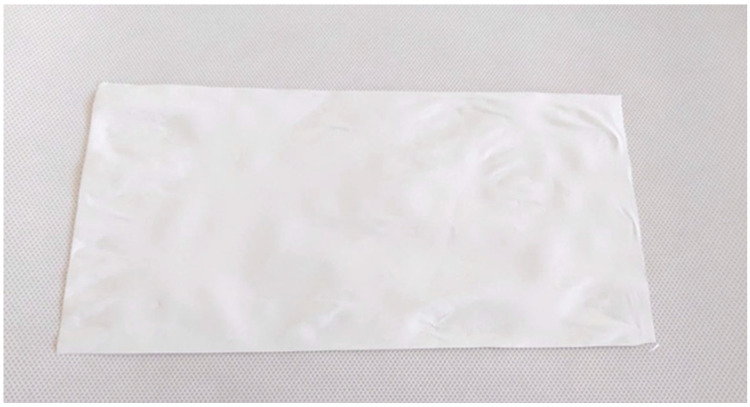
A sheet of polysulfone matrix membranes produced by electrospinning.

In this study, the main objective was to fabricate an anion-exchange membrane utilizing the electrospinning technique (Figure 4), employing readily available materials. The focus was to achieve final properties (conductivity and cell performance) comparable to those of our previously developed membrane based on the commercial Celgard membrane [20]. Several attempts have been made to determine the optimal processing parameters, which are shown in Table 1. The PSU-63 and PSU-77 membranes were produced on a baking paper substrate using a needle with a gauge of 18 (outer diameter: 1.27 mm). Both membranes were electrospun using the same polymer solution and electrospinning parameters as listed in Table 1. The only difference between the two samples was the deposition time, which resulted in different membrane thicknesses.

**Figure 5:**
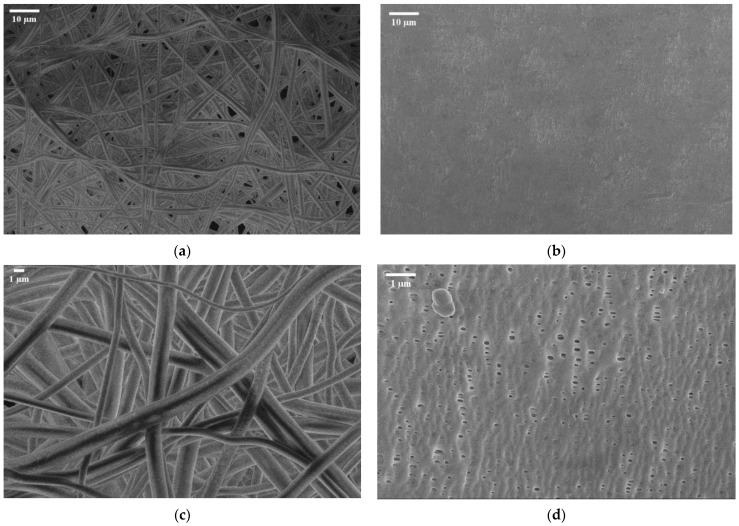
SEM image of electrospun polysulfone matrix (**a**) at M = 1 K and (**c**) at M = 3.5 K vs. SEM image of Celgard 3401 at (**b**) M = 1 k and (**d**) M = 10 K.

As demonstrated in the SEM image (Figure 5), the fabricated PSU electrospun membrane (left images) exhibits a randomly oriented fibrous network with a highly porous, nonwoven structure. The fibers appear interconnected, but the membrane lacks significant densification or alignment, while the Celgard 3401 membrane (right images) has a much more compact and uniform structure. The right lower-side image (higher magnification) shows elongated, slit-like pores, which are characteristic of Celgard’s microporous architecture. The upper right-side image (lower magnification) highlights its dense, smooth surface without visible fiber-like features, making it mechanically more robust (Table 2).

**Figure 6:**
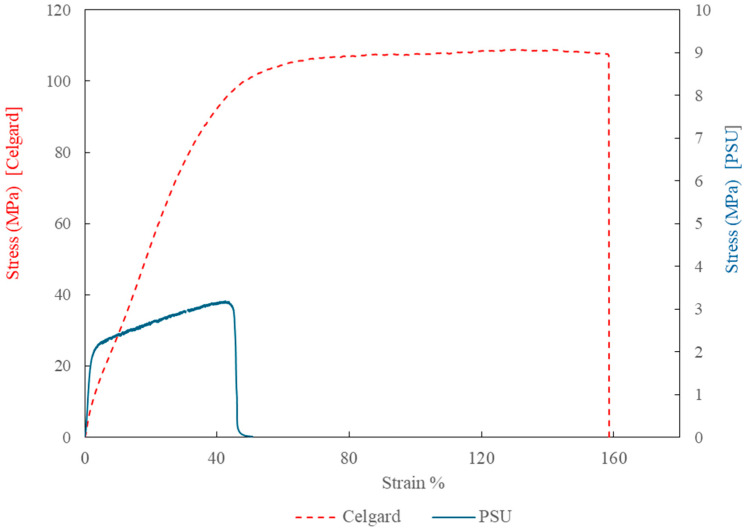
Stress–strain curves of the electrospun polysulfone membrane (solid blue line, E = 110.5 MPa) and the commercial Celgard 3401 membrane (dashed red line, E = 246.5 MPa), measured using a TA Instruments DMA850 at room temperature.

The PSU membranes in this study were tested in their native electrospun state without any post-processing or mechanical reinforcement. As a result, their mechanical performance reflects the intrinsic properties of the electrospun fibers and the as-formed membrane structure. In contrast, Celgard 3401 undergoes industrial processing, like stretching and densification, which improves its mechanical properties, making it stiffer and more resilient compared to the electrospun PSU membrane. Figure 6 shows the stress–strain curves of the electrospun polysulfone membrane and the commercial Celgard® 3401 membran. Notably, the activation of the PSU membrane with the Fumion ionomer solution leads to a substantial improvement in its mechanical properties. Specifically, the Young’s modulus increases by approximately 128% to 260%, and the tensile strength improves by 80% to 120%. However, this enhancement is accompanied by a significant reduction in ductility. Since this trade-off could potentially impact membrane handling and flexibility during integration into full-stack electrolyzer systems, it should be further investigated in future work to ensure suitability for practical implementation.

### 3.2. Anionic Composite Membrane Fabrication

Four different samples were produced through the procedure defined in Section 2.2. and are detailed in Table 3. The challenge in this methodology arises from inherent uncertainties at various stages, largely stemming from manual processes. Firstly, there is uncertainty in achieving reproducibility during solution preparation. The manual extraction of Fumion from the commercial solution introduces variability, exacerbated by inadequate control over environmental conditions such as humidity and temperature during drying. Secondly, uncertainty surrounds the measurement of sample thickness post-activation with Fumion. The activation process often induces wrinkling of the samples, leading to a non-uniform distribution of residual solution across the surface. This inconsistency results in a significant standard deviation in the thickness measurements across different sample points. These challenges underscore the need for enhanced process control and potentially automated procedures to mitigate variability and ensure more reliable experimental outcomes.

**Figure 7:**
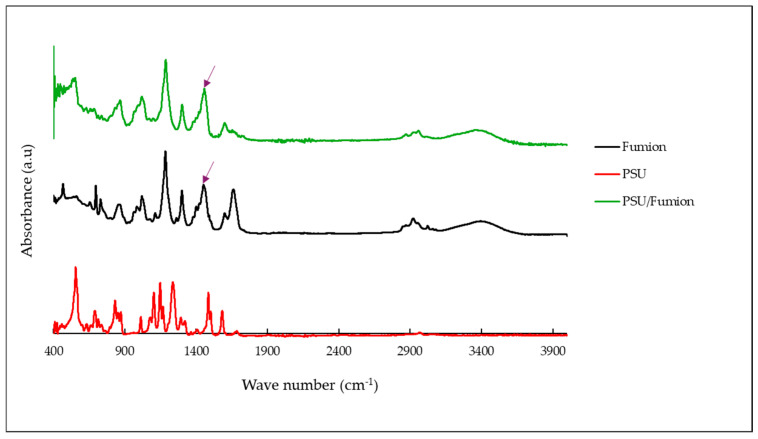
FTIR spectra of the PSU membrane before and after activation with the Fumion ionomer.

In the FTIR spectrum of the PSU/Fumion sample (Figure 7, green curve), new absorption bands appear that correspond to the characteristic peaks of the Fumion ionomer (black curve)—notably around 1470 cm^−1^ and 1340 cm^−1^—which are attributed to C–N stretching and CH_3_ bending vibrations of quaternary ammonium groups. These features are absent in the pristine PSU spectrum (red curve), confirming the presence of Fumion in the membrane structure after activation.

Among the prepared samples, the most promising result in terms of conductivity was observed with PSU-63 + Fu 15% (activated with Fumion solution at 15% *w*/*w*). Based on the results achieved, sample production and activation were repeated with 15% Fu solution additional times using the same procedure (Table 4). Subsequently, the best sample reproduced as S001 (PSU-77 + Fu 15%) underwent a long period cell test to evaluate the performance. Good and satisfactory results were obtained. Compared to Celgard, it achieved a higher maximum current density (1001 mAcm^−2^) and maintained this value consistently over 200 h of operation, which relates directly to the rate of electrochemical reactions, such as water splitting. The reference composite AEM with the Celgard backbone had a thickness of 39 µm (after activation), with the best conductivity measurement reaching 3 mS cm^−2^.

**Figure 8:**
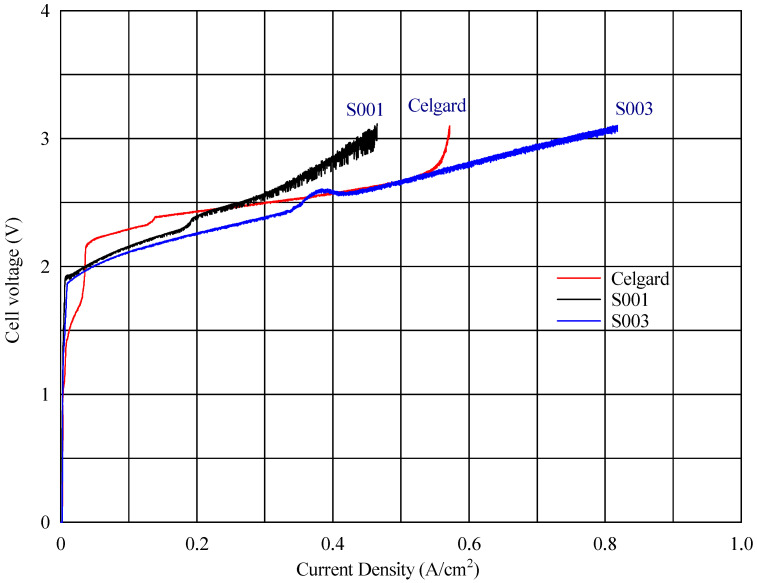
Cell voltage vs. current density profile at zero time for different AEMs.

**Figure 9:**
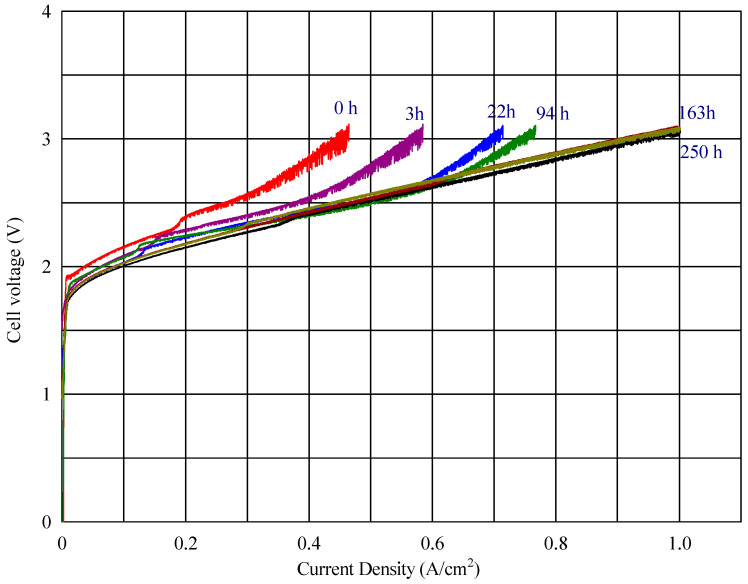
Cell voltage vs. current density profile for the membrane S001 at different times.

In the voltage–current density plot, as shown in Figure 6, at time zero, the cell with the fabricated membranes S003 and S001 reached a maximum current density of 818 mA cm^−2^ and 464 mA cm^−2^, respectively, while the commercial reference (composite AEM with Celgard) reached 569 mA cm^−2^ (Figure 8) Over time, the maximum current density of the cell with the new fabricated membrane (S001) increased progressively, reaching 1001 mA cm^−2^ (seen in Figure 9). This improvement suggests a conditioning or activation process within the cell, where the electrochemical environment stabilizes, allowing for better ion transport or reduced resistance.

**Figure 10:**
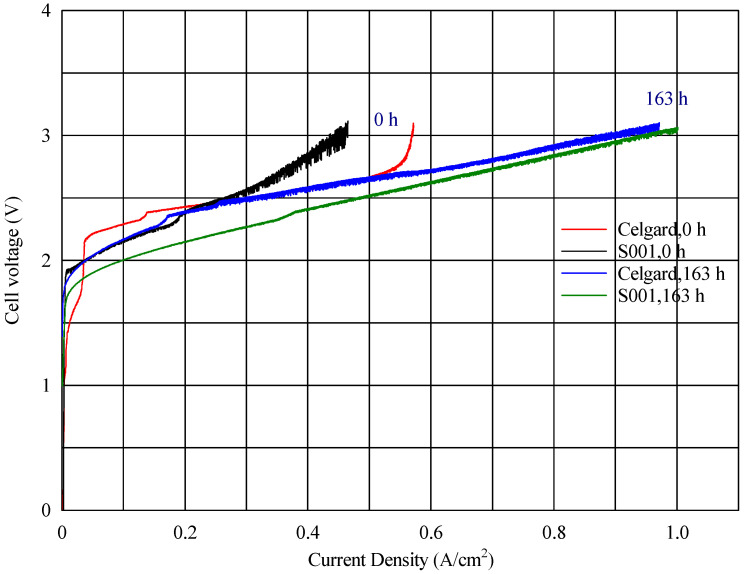
Comparing the V-I profiles of an electrospun polysulfone membrane (S001) and a Celgard 3401 membrane, both activated in the same manner with Fumion in the same electrolyzer.

To evaluate the membrane’s performance, the final sample was subjected to an extended cell test up to 250 h, yielding highly satisfactory results (Figure 10). The electrolysis voltage of the cells at a current density of 50 mA cm^−2^ during the operation time remained constant at 1.9 V, suggesting that the membrane maintained its ionic conductivity and structural integrity without significant degradation.

## 4. Conclusions

In this study, we successfully developed a composite anion-exchange membrane (AEM) using the electrospinning technique, demonstrating its potential for water electrolysis applications. By utilizing polysulfone as the base material and activating the membrane with a commercial quaternary ammonium-containing ionomer solution, we achieved ion conductivity comparable to our reference composite AEM, which was produced using the commercial Celgard membrane.

Our best-performing membrane is competitive against its commercial-based composite counterpart in terms of the maximum current density and long-term operational stability. However, its mechanical properties still require improvement. This study primarily focused on the electrochemical characteristics of the electrospun membranes, which, despite their lower mechanical strength, exhibited good ionic conductivity and overall electrochemical performance.

Future efforts should aim to optimize process standardization and introduce controlled activation techniques to improve membrane consistency and reproducibility. Since optimizing fabrication parameters in electrospinning is typically achieved through a systematic design of experiments (DOE), it would be particularly valuable to investigate the integration of machine learning or artificial intelligence tools to manage large-scale roll-to-roll production processes. These approaches can accelerate process optimization, quality control, and support the development of scalable, automated manufacturing workflows.

Overall, the innovations presented in this study contribute to the growing body of knowledge on electrospinning and its application in AEM development, which needs to be improved for further advancements in the commercialization of electrospun membranes, addressing both performance and cost effectiveness.

## Figures and Tables

**Table 1:** Electrospinning parameters of the PSU membrane.

Sample	Solution	FR (mL/h)	d (cm)	Vi (kV)	Vc (kV)	T (°C)	RH %	δ (µm)
PSU-63	PSU/NMP 24% *w*/*w*	2	15	+4	−15	22.7	52%	88
PSU-77	PSU/NMP 24% *w*/*w*	2	15	+5	−15	25	50%	120

**Table 2:** Comparison of mechanical properties of electrospun PSU membrane and commercial reference (both before activation).

Sample	Ave-δ (µm)	E (MPa)	UTS (MPa)
Celgard 3401	25	246.5	107.7
PSU-63	88	110.5	3.1

**Table 3:** Characteristics of four pieces of the same PSU membrane activated with different concentrations of Fumion solution in ethanol.

Sample	Activation Solution	W_0_ * (gr)	∆W	Air Permeability (cm^3^/cm^2^/s)	Ave-δ (µm)	δ-STDEV	σ (mS cm^−1^)	σ-STDEV
PSU-63 + Fu 5%	Fu 5%	0.0516	34.7%	0	100	8.07	0.3	0.001
PSU-63 + Fu 10%	Fu 10%	0.0542	72.3%	0	145	45.98	3.2	0.0723
PSU-63 + Fu 15%	Fu 15%	0.0540	198.5%	0	154.5	14.12	3.75	0.3478
PSU-63 + Fu 20%	Fu 20%	0.0548	239.2%	0	161	38.6	2.95	0.0362

* W_0_: initial weight before activation; ∆W: weight increase after activation.

**Table 4:** Characteristics of electrospun PSU membrane activated with Fumion solution at 15% *w*/*w*.

Sample	W_0_ (gr)	∆W	Air Permeability (cm^3^/cm^2^/s)	Ave-δ (µm)	δ-STDEV	σ (mS cm^−1^)	σ-STDEV
S003 (PSU-63 + Fu 15%)	0.0434	272%	0	162	30.72	3.02	0.0731
S001 (PSU-77 + Fu 15%)	0.0429	250%	0	150	27.54	4.85	0.1328

## Data Availability

Data are contained within the article.

## Abbreviations

The following abbreviations are used in this manuscript:

AEM	Anion-Exchange Membrane
PEM	Proton-Exchange Membrane
HEM	Hydroxide-Exchange Membrane
QA	Quaternary Ammonium
WE	Water Electrolysis
AEMWE	Anion-Exchange Membrane Water Electrolysis

## References

[1] Doshi J., Reneker D.H. (1995). Electrospinning process and applications of electrospun fibers. J. Electrost..

[2] Greiner A., Wendorff J.H. (2007). Electrospinning: A Fascinating Method for the Preparation of Ultrathin Fibers. Angew. Chem. Int. Ed. Engl..

[3] Huang Z.-M., Zhang Y.-Z., Kotaki M., Ramakrishna S. (2003). A review on polymer nanofibers by electrospinning and their applications in nanocomposites. Compos. Sci. Technol..

[4] Li D., Xia Y. (2004). Electrospinning of Nanofibers: Reinventing the Wheel?. Adv. Mater..

[5] Samsudin A.M., Hacker V. (2023). QPVA-Based Electrospun Anion Exchange Membrane for Fuel Cells. Int. J. Renew. Energy Dev..

[6] Yang J.M., Fan C.-S., Wang N.-C., Chang Y.-H. (2018). Evaluation of membrane preparation method on the performance of alkaline polymer electrolyte: Comparison between poly(vinyl alcohol)/chitosan blended membrane and poly(vinyl alcohol)/chitosan electrospun nanofiber composite membranes. Electrochim. Acta.

[7] Shang Z., Wycisk R., Pintauro P. (2021). Electrospun Composite Proton-Exchange and Anion-Exchange Membranes for Fuel Cells. Energies.

[8] Hibbs M.R., Fujimoto C.H., Cornelius C.J. (2009). Synthesis and Characterization of Poly(phenylene)-Based Anion Exchange Membranes for Alkaline Fuel Cells. Macromolecules.

[9] Xu T. (2005). Ion exchange membranes: State of their development and perspective. J. Membr. Sci..

[10] Liu Y., Pan Q., Wang Y., Zheng C., Wu L., Xu T. (2015). In-situ crosslinking of anion exchange membrane bearing unsaturated moieties for electrodialysis. Sep. Purif. Technol..

[11] Iravaninia M., Azizi S., Rowshanzamir S. (2017). A comprehensive study on the stability and ion transport in cross-linked anion exchange membranes based on polysulfone for solid alkaline fuel cells. Int. J. Hydrogen Energy.

[12] Sriram G., Dhanabalan K., Ajeya K.V., Aruchamy K., Ching Y.C., Oh T.H., Jung H.-Y., Kurkuri M. (2023). Recent progress in anion exchange membranes (AEMs) in water electrolysis: Synthesis, physio-chemical analysis, properties, and applications. J. Mater. Chem. A.

[13] Arges C.G., Ramani V. (2012). Alkaline Stability and Ion Conductivity of Polysulfone Anion Exchange Membranes (AEMs) with Different Cation Chemistries. ECS Meet. Abstr..

[14] Das G., Choi J.-H., Nguyen P.K.T., Kim D.-J., Yoon Y.S. (2022). Anion Exchange Membranes for Fuel Cell Application: A Review. Polymers.

[15] Du N., Roy C., Peach R., Turnbull M., Thiele S., Bock C. (2022). Anion-Exchange Membrane Water Electrolyzers. Chem. Rev..

[16] Krivina R.A., Lindquist G.A., Yang M.C., Cook A.K., Hendon C.H., Motz A.R., Capuano C., Ayers K.E., Hutchison J.E., Boettcher S.W. (2022). Three-Electrode Study of Electrochemical Ionomer Degradation Relevant to Anion-Exchange-Membrane Water Electrolyzers. ACS Appl. Mater. Interfaces.

[17] Razmjooei F., Morawietz T., Taghizadeh E., Hadjixenophontos E., Mues L., Gerle M., Wood B.D., Harms C., Gago A.S., Ansar S.A. (2021). Increasing the performance of an anion-exchange membrane electrolyzer operating in pure water with a nickel-based microporous layer. Joule.

[18] Kim M., Lee D., Qi M., Kim J. (2024). Techno-economic analysis of anion exchange membrane electrolysis process for green hydrogen production under uncertainty. Energy Convers. Manag..

[19] Giovanelli A., Pozio A., Pucci A., Geppi M., Martini F. (2024). Fumasep FAA-3-PK-130: Exploiting multinuclear solid-state NMR to shed light on undisclosed structural properties. Polymer.

[20] Rakhshani S., Araneo R., Pucci A., Rinaldi A., Giuliani C., Pozio A. (2023). Synthesis and Characterization of a Composite Anion Exchange Membrane for Water Electrolyzers (AEMWE). Membranes.

[21] Theron S., Zussman E., Yarin A. (2004). Experimental investigation of the governing parameters in the electrospinning of polymer solutions. Polymer.

[22] Luo C., Nangrejo M., Edirisinghe M. (2010). A novel method of selecting solvents for polymer electrospinning. Polymer.

[23] Luo C.J., Stride E., Edirisinghe M. (2012). Mapping the Influence of Solubility and Dielectric Constant on Electrospinning Polycaprolactone Solutions. Macromolecules.

[24] Rošic R., Pelipenko J., Kocbek P., Baumgartner S., Bešter-Rogač M., Kristl J. (2012). The role of rheology of polymer solutions in predicting nanofiber formation by electrospinning. Eur. Polym. J..

[25] Chen H., Elabd Y.A. (2009). Polymerized Ionic Liquids: Solution Properties and Electrospinning. Macromolecules.

[26] Yang Y., Jia Z., Li Q., Guan Z. (2006). Experimental investigation of the governing parameters in the electrospinning of polyethylene oxide solution. IEEE Trans. Dielectr. Electr. Insul..

[27] Fong H., Chun I., Reneker D.H. (1999). Beaded nanofibers formed during electrospinning. Polymer.

[28] Fiaschini N., Giuliani C., Vitali R., Tammaro L., Valerini D., Rinaldi A. (2022). Design and Manufacturing of Antibacterial Electrospun Polysulfone Membranes Functionalized by Ag Nanocoating via Magnetron Sputtering. Nanomaterials.

[29] Rakhshani S., Araneo R., Rinaldi A., Pozio A. Electrospinning of Polysulphone to Fabricate Anion Exchange Membrane for Cost-Effective Water Electrolysis. Proceedings of the 2023 IEEE International Conference on Environment and Electrical Engineering and IEEE Industrial and Commercial Power Systems Europe, EEEIC/I and CPS.

[30] Pozio A., Cemmi A., Carewska M., Paoletti C., Zaza F. (2010). Characterization of gas diffusion electrodes for polymer electrolyte fuel cells. J. Fuel Cell Sci. Technol..

